# The inhibitory effect and mechanism of Yi-qi-hua-yu-jie-du decoction on the drug resistance of gastric cancer stem cells based on ABC transporters

**DOI:** 10.1186/s13020-022-00647-y

**Published:** 2022-08-09

**Authors:** Wenjie Huang, Fang Wen, Peixing Gu, Jiatong Liu, Yun Xia, Ye Li, Jiayu Zhou, Siyuan Song, Shuai Ruan, Suping Gu, Xiaoxue Chen, Peng Shu

**Affiliations:** 1 Department of Oncology, Affiliated Hospital of Nanjing University of Chinese Medicine, 155 Hanzhong Road, Nanjing, 210000 Jiangsu China; 2 First College of Clinical Medicine, Nanjing University of Chinese Medicine, Nanjing, China; 3 Department of Respiratory, Wujin Hospital of Traditional Chinese Medicine, Changzhou, China

**Keywords:** Gastric cancer, Drug resistance, Stem cells, Yi-qi-hua-yu-jie-du decoction, ABC transporters, PI3K/Akt/Nrf2 pathway

## Abstract

**Background:**

The drug resistance of tumor stem cells is an obstacle in gastric cancer (GC) treatment and the high expression of ABC transporters is a classic reason for drug resistance. This study aimed to construct a reliable GC drug-resistant stem cell model and explore the inhibitory effect and mechanism of Yi-qi-hua-yu-jie-du medicated serum (YQHY) on the drug resistance of GC stem cells based on ABC transporters.

**Methods:**

The tumor stemness biomarker CD44 was primary identification from WGCNA. The magnetic-activated cell sorting (MACS) method was used to separate CD44( +)BGC823/5-Fu (BGC823/5–Fu-CSCs) cells and the stemness characteristics were verified from multiple dimensions. Then, the drug resistance index and expression of ABC transporter genes MDR1 and MRP1 were detected in CD44(−)/CD44(+) cells. The inhibition and apoptosis rates of the cells administrated with YQHY or/and 5-Fu were calculated to confirm that YQHY can suppress the drug resistance of BGC823/5-Fu-CSCs. Afterwards, the effects of YQHY on the expression of MDR1 and MRP1 and the activation of the PI3K/Akt/Nrf2 pathway were observed. Finally, under the administration of IGF-1 (the activator of PI3K/Akt pathway) and Nrf2 siRNA, the mechanism of YQHY on reversing the drug resistance of BGC823/5–Fu-CSCs through inhibiting the expression of MDR1 and MRP1 via PI3K/Akt/Nrf2 was verified.

**Results:**

CD44 was a reliable GC stemness biomarker and can be applied to construct the drug-resistant GC stem cell model CD44(+)BGC823/5-Fu. The growth rate, cell proliferation index, soft agar colony formation, expression of stemness specific genes and tumorigenesis ability of CD44(+)BGC823/5-Fu cells were significantly higher than those of CD44(−)BGC823/5-Fu cells. BGC823/5–Fu-CSCs exhibited strong drug resistance to 5-Fu and high expression of ABC transporter genes MDR1 and MRP1 compared to CD44(-) cells. YQHY increased the inhibition and apoptosis rates to efficiently inhibit the drug resistance of BGC823/5–Fu-CSCs. Meanwhile, it suppressed the expression of MDR1 and MRP1 and restrained the activation of PI3K/Akt/Nrf2 signaling pathway. Finally, it was found that IGF-1 partially restored the activation of PI3K/Akt/Nrf2 pathway, alleviated the inhibition of MDR1 and MRP1, blocked the proliferation-inhibitory and apoptosis-promotion effects. YQHY and si-Nrf2 synergistically suppressed the MDR1/MRP1 expression and the drug resistance of BGC823/5–Fu-CSCs.

**Conclusions:**

CD44 was a reliable GC stemness biomarker, and the high expression of ABC transporter genes MDR1 and MRP1 was an important feature of drug-resistant stem cells. YQHY inhibited the MDR1 and MRP1 expression via PI3K/Akt/Nrf2 pathway, thus reversing the drug resistance of BGC823/5–Fu-CSCs.

**Supplementary Information:**

The online version contains supplementary material available at 10.1186/s13020-022-00647-y.

## Background

Gastric cancer is a common malignant tumor and is the third leading cause of cancer-related death worldwide [1]. Chemotherapy is the major therapeutic method for GC, and the overall survival rate has been improved with the progress of new drugs and regimens. However, the recurrence and metastasis rates of GC are still relatively high, and are closely related to the drug resistance in cancer treatment.

Previous studies have confirmed that these phenomena are associated with the existence of cancer stem cells (CSCs) [2]. The CSC theory was initially proposed by Moore et al. in 1973 [3], and CSCs were first identified in acute myeloid leukemia [4]. Many studies have revealed that CSCs, which can be formed by a small number of drug-resistant cells that are not cleared by chemotherapy, manifest powerful tumorigenesis ability and self-renewal capacity. CSCs usually exhibit a strong feature of drug resistance and leave seeds for tumor recurrence and metastasis [5]. In recent years, a variety of surface markers, such as aldehyde dehydrogenase, CD133, CD13, CD24 and CD44, have been discovered [6]. And the high expression of these specific biomarkers in stem cells was closely associated with an increase tendency of tumor drug resistance.

Besides, the high expression of ATP2 binding cassette transporters (ABC transporters) in CSCs is an important reason for GC drug resistance [7]. In a variety of tumor types, including gastrointestinal cancer, the high expression of ABC transporter genes (mainly including MDR1/P-gp, MRP1) can drain chemotherapy drugs out of cancer cells, leading to drug resistance, thus accelerating cell survival and tumor recurrence [8]. Alisi [9] suggested that the over-expression of ABC transporters was probably the most critical reason for the chemotherapeutic drug resistance in CSCs. In terms of mechanistic studies, it has been found that ABC transporters affect tumor drug resistance through multiple signaling pathways, such as PI3K/Akt [10], NF-κB [11], Nrf2 [12], Wnt/β-catenin [13], JNK [14].

Although numerous studies have revealed the importance of targeting CSCs drug resistance in improving tumor treatment effectiveness, there is still a lack of safe and effective drug resistance reversal agents in clinical practice. It is of certain research significance to find efficient inhibitors that targeting CSCs drug resistance in GC. Recently, traditional Chinese medicine, characterized by multiple targets and low toxicity, has gradually become a hot topic in tumor treatment. Yi-qi-hua-yu-jie-du decoction was created by the famous National Chinese Medicine Practitioner Professor Shenlin Liu. It was modified from the classic Chinese medicine compound “Gui-shao-liu-jun-zi Decoction” (an ancient prescription for the treatment of spleen and stomach diseases including gastric cancer originated from “Bi Hua Yi Jing”). The outstanding effect of Yi-qi-hua-yu-jie-du decoction in prolonging the disease-free survival period of GC patients was proved by the State Administration of Traditional Chinese Medicine multi-center clinical research program (NO. 200807022) (taking 8 years to follow up 489 patients) [15]. Our research team has conducted several basic studies on Yi-qi-hua-yu-jie-du decoction and revealed its efficacy in reversing drug resistance in GC [16, 17].

**Fig. 1:**
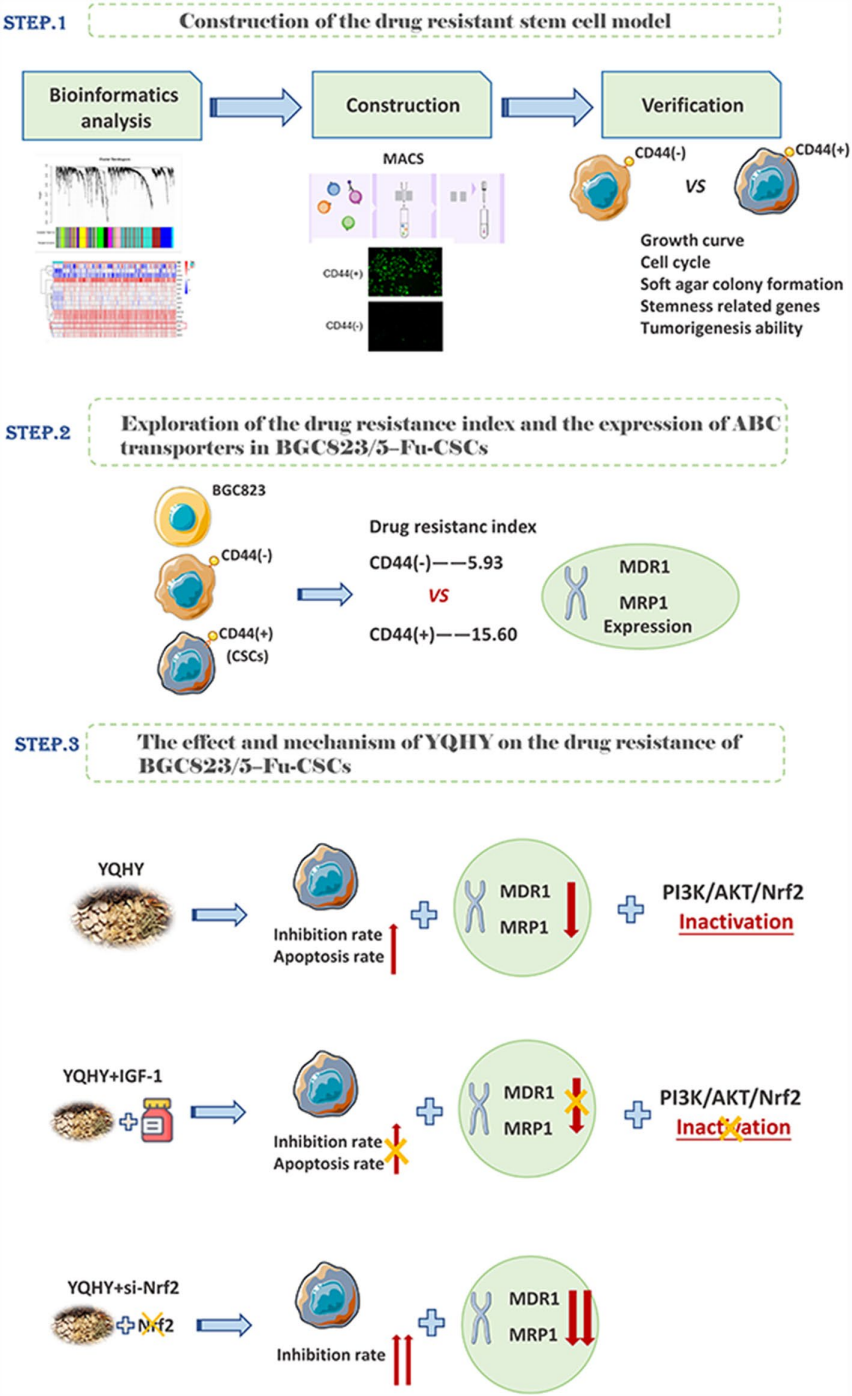
The main procedure of this research

In this study, we established a GC drug-resistant stem cell model (BGC823/5–Fu-CSCs) and found these cells exhibited high expression of ABC transporter genes (MDR1 and MRP1). Subsequently, we explored the suppressive effect and mechanism of Yi-qi-hua-yu-jie-du decoction medicated serum on the drug resistance of BGC823/5–Fu-CSCs regulated by ABC transporters. The research procedure was shown in Fig. 1.

## Materials and methods

### Cell lines and main regents

The human gastric cancer cell line BGC823 was purchased from KeyGEN BioTECH Co., Ltd. (China).

The drug-resistant cell line BGC823/5-Fu was established by the low-dose multiple shock method in our previous research [17] and stored at -196 °C. The drug resistance index of BGC823/5-Fu was 13.

The serum-free medium (SFM) was composed of 95% DMEM/F12 (Gibco, USA), 1% 2 µg/ml EGF (Peprotech, USA), 1% 2 µg/ml bFGF (Peprotech, USA), 2% B27 (Gibco, USA), 1% 100 U/ml penicillin/streptomycin (Gibco, USA) and 0.4 U insulin (Sigma, USA). The IGF-1 was purchased from Sigma (USA). The P-gp, MRP1, PI3K, p-PI3K, AKT, p-AKT, Nrf2 and β-actin antibodies were purchased from Bioss, Inc. (China) and Proteintech Group, Inc. (USA). HRP-labeled Goat Anti-Rabbit IgG, HRP-labeled Goat Anti-Mouse IgG and Alexa Fluor 488-labeled Goat Anti-Rabbit IgG were purchased from Beyotime Biotechnology (China). The Annexin V-FITC/PI Kit was purchased from KeyGEN BioTECH Co., Ltd. (China). CD44 MicroBead Kit was purchased from Miltenyi Biontec. (Germany). Lipofectamine2000 was purchased from Invitrogen (USA).

### Preparation and quality control of Yi-qi-hua-yu-jie-du decoction

**Fig. 2:**
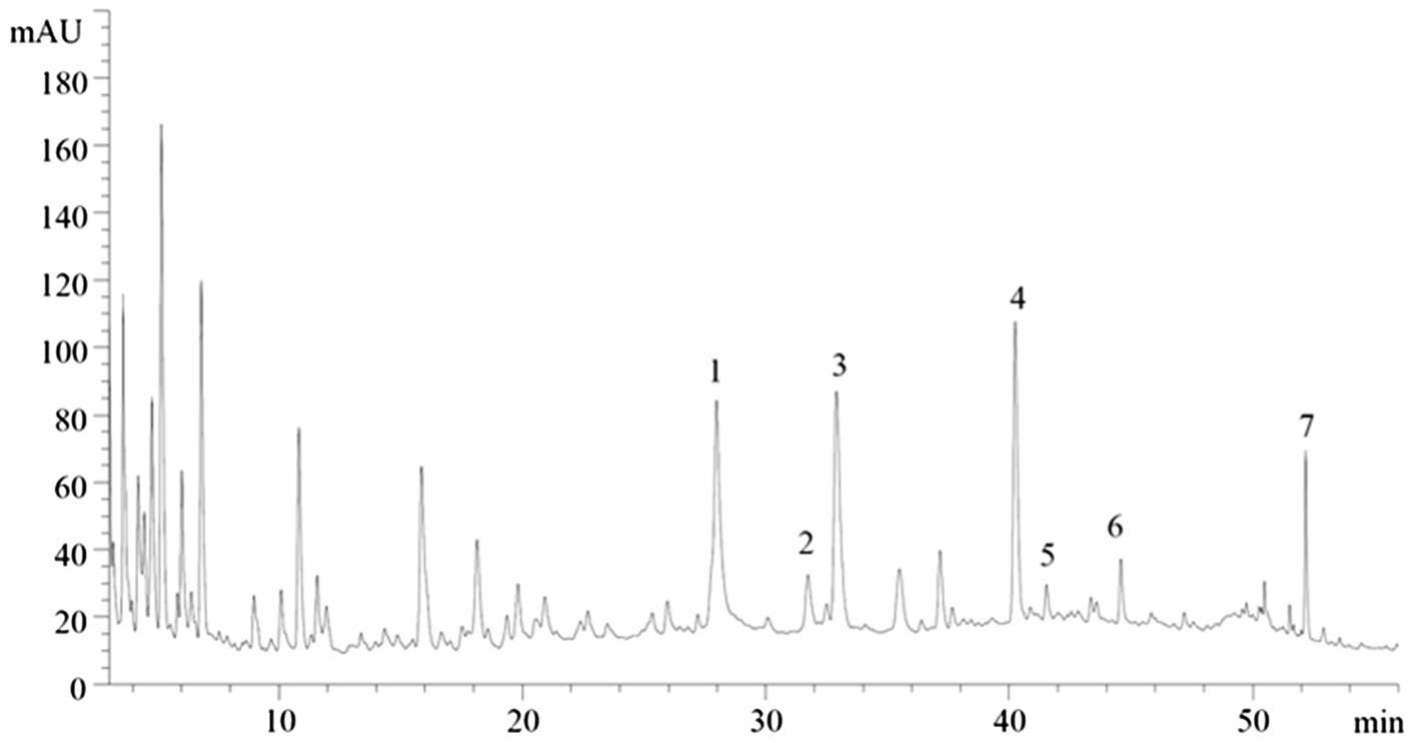
The HPLC chromatogram of Yi-qi-hua-yu-jie-du decoction. 1.Paeoniflorin; 2.Ferulic acid; 3.Rutin; 4.Hesperidin; 5.Rosmarinic acid; 6.Salvianolic acid; 7.Glycyrrhizic acid

Yi-qi-hua-yu-jie-du decoction was composed of 15 Chinese herbal medicines (Additional file 1: Supplementary Table 1). The herbal medicine materials were purchased from the pharmacy department in the Affiliated Hospital of Nanjing University of Chinese Medicine, and the medicinal material quality was identified by professional Chinese pharmacists. Then, the water solution of these herbs was prepared at a concentration of 2.5 g/ml. The quality control was conducted by high-performance liquid chromatography (HPLC). According to the Chinese Pharamacopoeia (2015 Edition), paeoniflorin (1), ferulic acid (2), rutin (3), hesperidin (4), rosmarinic acid (5), salvianolic acid (6) and glycyrrhizic acid (7) were quantified. The pharmic concentrations of these standard substances were 552.7 μg/ml, 60.9 μg/ml, 310.7 μg/ml, 904.2 μg/ml, 61.4 μg/ml, 214.1 μg/ml, and 396.2 μg/ml, respectively (Fig. 2). Standard reagents were purchased from Chengdu Must Bio-technology Co., Ltd. (China).

### Preparation of Yi-qi-hua-yu-jie-du medicated serum (YQHY)

Twenty Sprague–Dawley rats (200–220 g) were randomly divided into the blank group and the YQHY group. Mice in each group were intragastrical given Yi-qi-hua-yu-jie-du decoction physic liquor or normal saline once a day (dose = clinical usual dosa × animal equivalent area coefficient) according to the body weight (10 ml/kg) for 14 days. One hour after the last administration, blood was taken from the abdominal aorta and centrifuged (3000 rpm, 10 min) to separate the serum. The serum was stewed in water at 56 °C for 30 min, filtered with a 0.22 µm cellulose acetate membrane and stored at -80 °C. In this study, 4%, 8% and 12% of the serum was applied as the low, median, and high YQHY groups, respectively (the blank serum was used to make the total serum content of each group to be 12%).

### Bioinformatics studies

Transcriptome data and clinical data were obtained from TCGA (https://portal.gdc.cancer.gov/). The mRNAsi and EREG-mRNAsi indices were acquired from Phi LTH’s research [18]. Kaplan–Meier Plotter (https://kmplot.com/analysis/) was used to evaluate the prognostic value of drug resistance genes in GC patients.

The beeswarm package in R4.0.4 was applied to compare the mRNAsi index, and the limma package was used to identify the differentially expressed genes (DEGs) (|logFC|> 2.0 and *P* < 0.05). A co-expression network targeting DEGs was constructed through the WGCNA package. To assess the significance of each module, we calculated the gene significance (GS) and analyzed the interaction between the levels of gene expression and sample characteristics. The calculation of GS was the log10 conversion of the p-value in the linear regression between gene expression and mRNAsi or EREG-mRNAsi. In addition, the mean GS within the module was defined as module significance (MS), which was determined to analyze the link between each module and sample features. And the module with the largest MS was considered as the module that was strongly related to the sample characteristic. Finally, the GS and module membership (MM, relationship between genes in the given module and the expression profiles) were set as the thresholds for identifying the key genes in the module with GS > 0.5 and MM > 0.8. The process of data clustering and module construction was shown in Additional file 1: Supplementary Figure 1.

### Magnetic activated cell sorting (MACS) method

BGC823/5-Fu cells were resuspended in 45 µl MACS buffer, and the cell density was adjusted to 1 × 10^6^/ml. Then, 20 µl CD44 magnetic beads and 20 µl Fcr blocking agent were added to the cells. The cells were incubated at 4 °C for 15 min, resuspended in 500 µl MACS buffer and added to the LS column in the magnetic field with a MACS separator. The outflow suspension containing CD44(-)BGC823/5-Fu cells was collected. Then, the LS column was removed from the MACS separator and washed with MACS buffer. CD44( +)BGC823/5-Fu cells in the eluent were collected for the following experiments.

### Flow cytometry

Cells were seeded in six-well plates at 2 × 10^5^ cells per well and exposed to 5-Fu/YQHY for 48 h, and Annexin-V/PI staining was added in sequence according to the manufacturer’s instruction book. Flow cytometry detection was applied for apoptosis or cell cycle analysis.

### Soft agar assay

1 × 10^4^ CD44(-)BGC-823/5-Fu or CD44( +)BGC-823/5-Fu cells were suspended in 0.3% agar conventional medium and cultured in 0.5% agar medium at 37 °C for 2 weeks. The number and diameter of colonies formed were observed with an inverted microscope.

### CCK8 assay

Cells were seeded in 96-well plates and incubated with 5-Fu and/or YHQY at different concentrations for 48 h. 10 µl Cell™ Counting Kit-8 (Beyotime Biotechnology, China) was added, and the cells were incubated for 1 h at 37 °C avoid light. The absorbance was measured at 450 nm using a microplate reader.

### Immunofluorescence staining

Cells were incubated in petri dishes containing glass slides overnight. The glass slides were fixed with 4% paraformaldehyde for 30 min. Then, the cells were permeabilized with 0.1% Triton X-100 for 20 min. The slides were blocked with 5% BSA for 1 h. The slides were incubated with the primary antibody for 12 h. Then, the slides were incubated with fluorescent secondary antibody for 1 h and stained with Hoechst33342 for 10 min. The protein expression status was observed under a fluorescent microscope.

### Q-PCR

Cells were collected for the extraction of total RNA, and reverse transcription was conducted using HiscriptIII qRT SuperMix for qPCR (Vazyme, China). ChamQ Universal SYBR qPCR Master Mix (Vazyme, China) was applied for quantitative real-time polymerase chain reaction (Q-PCR) detection. Primer sequences were listed in Additional file 1: Supplementary Table 2.

### Western blot

The cell protein was extracted, and the concentration was determined by BCA assay. The protein was separated by SDS–PAGE and transferred to PVDF membranes. The membranes were blocked with 5% bovine serum albumin for 1 h, incubated with the primary antibody overnight at 4 °C and then incubated with the secondary antibody for 1 h. Enhanced chemiluminescence reagent was used to obtain images.

### In vivo tumor formation experiment

The nude mice were purchased from Qinglongshan Animal Breeding Farm, Inc. (China) (male; 7 weeks old; 18–20 g weight). Each mice received subcutaneous injection of 1 × 10^5^ CD44(-)BGC-823/5-Fu or CD44( +)BGC-823/5-Fu cells. The tumor volume was measured on day 1,3,5,7,9,11,14. The nude mice were sacrificed on day 14 and the tumor volume/weight were measured.

### Si-RNA transfection

Human Nrf2 siRNA/si-NC were purchased from Genomeditech (China). Si-Nrf2 or si-NC was transfected into BGC823/5-Fu-CSCs cells using Lipofectamine2000, incubated in Opti-MEN for 6 h. The expression of Nrf2 after transfection was determined by Western blot.

### Statistical analysis

SPSS 25.0 software was used for statistical analysis. Independent-samples T test was used for the comparison between two groups, and one-way ANOVA was used for the comparison between multiple groups. *^(#/△)^*P*  < 0.05, **^(##/△△)^*P* < 0.01 were considered to be statistically significant.

## Resullts

### The primary identification of tumor stemness biomarkers through bioinformatics analysis

In order to screen out an effective biomarker to construct the drug-resistant stem cell model, we preliminarily identified the stemness related genes in GC through the bioinformatics analysis.

**Fig. 3:**
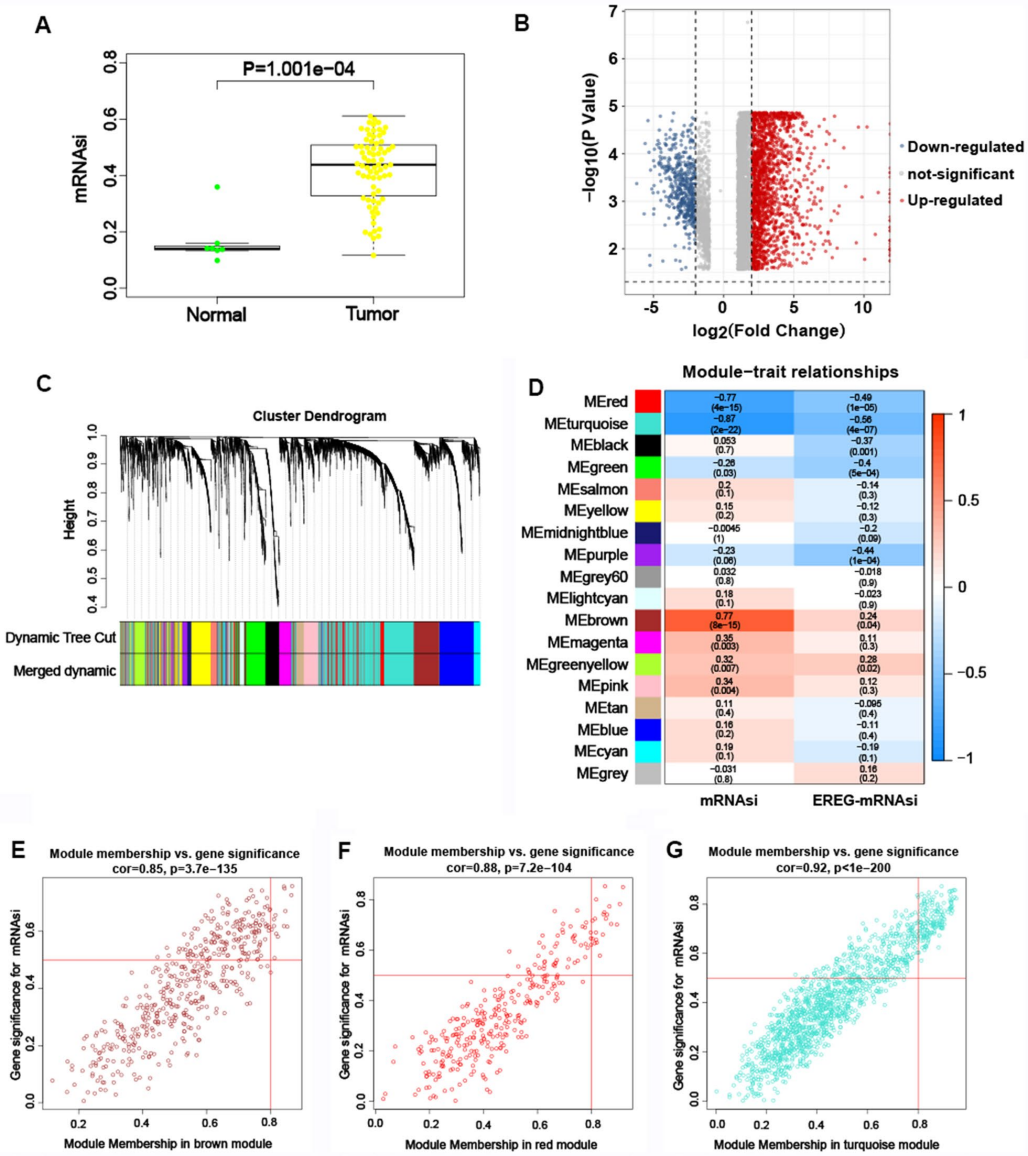
The mRNAsi and DEGs in GC and the WGCNA. **A** Differences in the expression of mRNAsi in GC samples compared to non-tumor samples. **B** Volcano plot demonstrated the differential expression in GC samples compared to non-tumor samples. **C** Co-expression module identification in GC. **D** Heatmap showed the association and significant differences between the gene modules and mRNAsi scores/EREG-mRNAsi. The correlation coefficient was shown in the upper row in each module, and the *P* value was shown in the bracket. Scatter plot of module eigengenes in the brown (**E**), red (**F**) and turquoise (**G**) module

The mRNAsi can reflect the tendency of tumor cell dedifferentiation and is a potential marker in CSCs identification. A remarkably higher mRNAsi was found in GC samples than in non-tumor samples (Fig. 3A). We used RNA-seq data from TCGA to identify 1572 DEGs (1225 upregulated and 347 downregulated) which can regulate the stemness of GC cells (Fig. 3B).

**Fig. 4:**
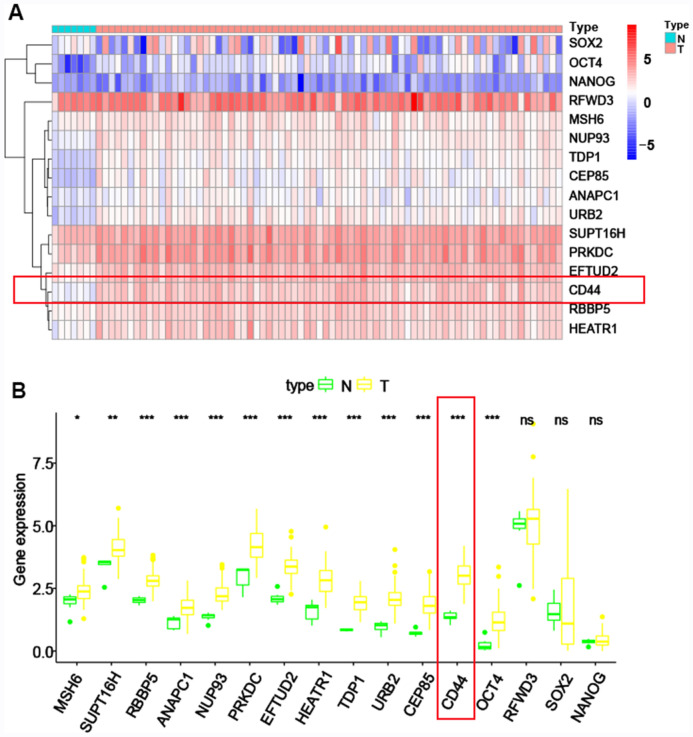
The expression difference of the key genes in GC. **A** The heatmap of the key genes in GC samples and non-tumor samples. **B** The boxplot of the key genes in GC samples and non-tumor samples. **A**, **B** CD44 exhibited a higher expression in GC samples compared to no-tumor samples

To identify the gene modules with biological significance and search for genes strongly linked to GC stemness, we constructed a gene co-expression network by the weighted gene co-expression network analysis (WGCNA). 18 modules were constructed (Fig. 3C). The red, turquoise and brown modules were strongly correlated with the stemness of GC according to the MS and R^2^ (Fig. 3D). The brown module was positively correlated with mRNAsi (R^2^ = 0.77, *P* = 8e−15) (Fig. 3E), while the red and turquoise modules exhibited a negative correlation with mRNAsi (R^2^ =  − 0.77, *P* = 4e−15; R^2^ =  − 0.87, *P* < 2e−22) (Fig. 3F, 3G). Therefore, the brown module was further analyzed to search for the key stemness genes of GC. Finally, 16 important stemness genes (MSH6, SUPT16H, RBBP5, ANAPC1, NUP93, PRKDC, EFTUD2, HEATR1, TDP1, URB2, CEP85, CD44, OCT4, RFWD3, SOX2 and NANOG) were identified with the thresholds of MM > 0.8 and GS > 0.5. The heatmap (Fig. 4A) and boxplot (Fig. 4B) were drawn to demonstrate the expression of these genes in GC samples and CD44 was remarkably upregulated.

Based on the above analysis, we searched relevant literature and found that CD44, as a classic tumor stemness related gene and a common marker on the tumor cell surface, was considered to be a reliable GC stem cell sorting phenotype [19, 20]. Other studies have shown that CD44 was intimately related to drug resistance in tumors [21, 22]. Therefore, we selected CD44 as a biomarker to establish a drug-resistant stem cell model of gastric cancer for further research.

### The construction and verification of GC drug-resistant stem cell model BGC823/5–Fu-CSCs

**Fig. 5:**
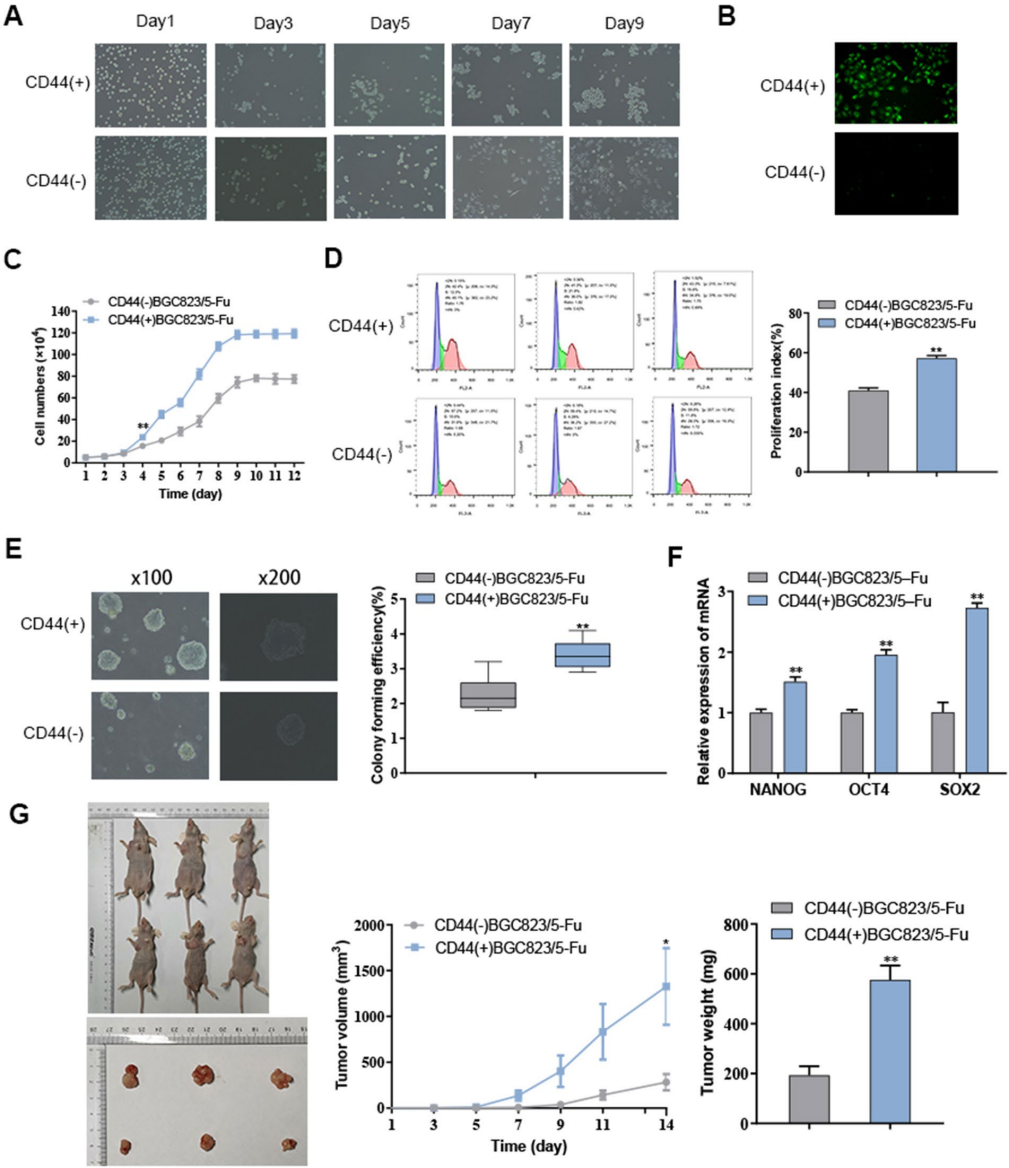
The separation and verification of CD44(+)BGC823/5-Fu stem cells. The growth status (**A**), fluorescence staining observation (**B**), cell growth curve (**C**), cell cycle (**D**) and colony formation analysis (**E**) of CD44(+)BGC823/5-Fu and CD44(-)BGC823/5-Fu cells. (**F**) The expression of stemness-related genes in CD44(+)BGC823/5-Fu and CD44(−)BGC823/5-Fu cells. (**G**) The tumor volume and weight of CD44(+)BGC823/5-Fu and CD44(-)BGC823/5-Fu-inoculated nude mice

CD44( +)BGC823/5-Fu cells were isolated using the MACS method, seeded in ultralow adhesion six-well plates, and routinely cultured in serum-free medium (SFM). Morphological observation showed that CD44( +)BGC823/5-Fu cells tended to grow in suspension and aggregate into grape clusters. After 5 days, the cells aggregated into spheroids. After 7 days, the cell spheroid size increased rapidly, and the cells were tightly bound to each other. While CD44(-)BGC823/5-Fu cells were hard to aggregate into spherical growth (Fig. 5A). The cell smears of CD44(+)BGC823/5-Fu and CD44(-)BGC823/5-Fu were incubated with FITC-CD44 antibody (1:1000) for 30 min. And the fluorescence microscopy observation showed that CD44( +)BGC823/5-Fu cells demonstrated high expression of green fluorescence, which indicated that the cells can maintain the CD44( +) phenotypic characteristics during the differentiation process (Fig. 5B). In addition, the cell growth curve exhibited that the proliferation ability of CD44( +)BGC823/5-Fu cells was significantly stronger than that of CD44(-)BGC823/5-Fu cells (*P* < 0.01 since the day 4) (Fig. 5C).

Flow cytometry was applied to detect the cell cycle of CD44( +)BGC823/5-Fu and CD44(-)BGC823/5-Fu cells. The cell proliferation index [PI = (S + G2/M)/(G0/G1 + S + G2/M)*100%] of the two cell lines was 57.19 ± 1.37% and 40.98 ± 1.31%, respectively (*P* < 0.01), also indicating that CD44( +)BGC823/5-Fu gained stronger proliferative capacity than CD44(-)BGC823/5-Fu (Fig. 5D). The soft agar assay showed that CD44( +)BGC823/5-Fu formed more colonies and shaped larger colony diameters. The colony formation rate was 3.40 ± 0.43, which was higher than 2.27 ± 0.50 of CD44(-)BGC823/5-Fu (*P* < 0.01) (Fig. 5E). In addition, we analyzed the differences in stemness related gene expression in the two cell lines. The expression of NANOG, OCT4 and SOX2 in CD44( +)BGC823/5-Fu cells was significantly higher than that in CD44(-)BGC823/5-Fu cells (*P* < 0.01) (Fig. 5F). Besides, a tumor formation experiment in nude mice revealed that the tumor volume (*P* < 0.05) and weight (*P* < 0.01) of CD44( +)BGC823/5-Fu inoculated mice were conspicuously larger than those of CD44(-)BGC823/5-Fu inoculated mice (Fig. 5G).

Based on the above experiments, we successfully constructed a CD44( +) gastric cancer stem cell model and confirmed the stemness characteristics from multiple dimensions, revealing that CD44(+)BGC823/5-Fu was a reliable GC stem cell model (we named it “BGC823/5–Fu-CSCs”).

### BGC823/5–Fu-CSCs exhibited strong drug resistance to 5-Fu and high expression of ABC transporter genes

**Fig. 6:**
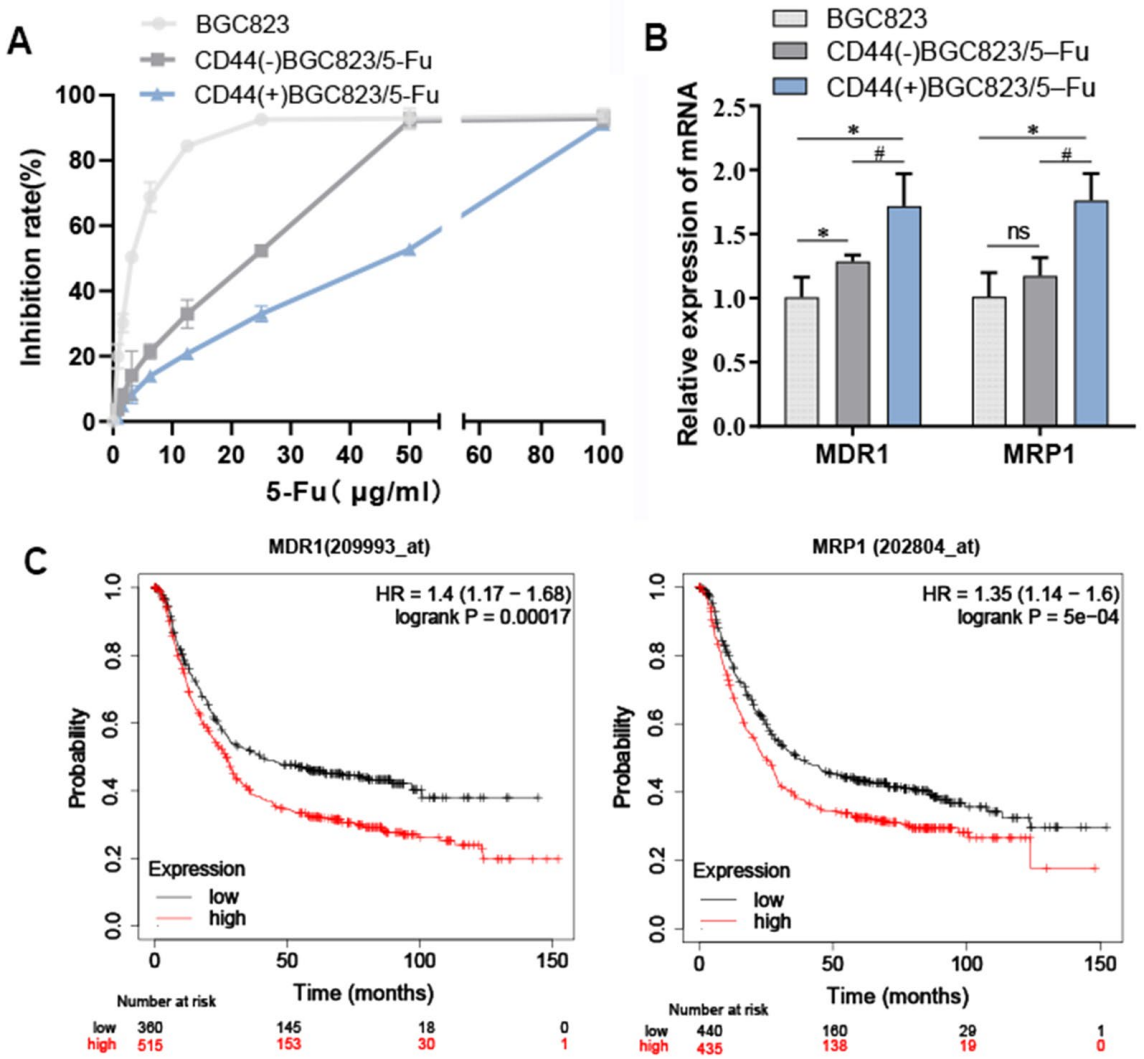
The sensitivity of CD44(−) and CD44(+)(CSCs) cells to 5-Fu and the gene expression and prognostic value of ABC transporters. **A** The inhibition rate of BGC823, CD44(-)BGC823/5-Fu and CD44(+)BGC823/5-Fu (BGC823/5-Fu-CSCs) treated with 5-Fu after 48 h. **B** The gene expression of MDR1 and MRP1 in the three cell lines detected by Q-PCR. **C** The Kaplan–Meier curve related to OS of MDR1 and MRP1 in GC patients

To explore the drug resistance degree of cells with different CD44 expression status, the CCK8 assay was used to detect the sensitivity of BGC823, CD44(-)BGC823/5-Fu and CD44( +)BGC823/5-Fu to 5-Fu, and the drug resistance index of CD44(-) cells and CD44( +) cells were 5.93 and 15.60, respectively (Fig. 6A). It suggested that BGC823/5–Fu-CSCs cells (marked with CD44 positive) presented stronger drug resistance to 5-Fu. Since the existence of ABC transporters is one of the classic mechanisms of chemotherapy resistance. It acts as a "transport pump" to pump chemotherapy drugs out of the cells. We detected the expression of MDR1 and MRP1 (two important ABC transporter genes) in BGC823, CD44(−)BGC823/5-Fu and CD44(+)BGC823/5-Fu cells. It demonstrated that CD44(+) cells showed higher expression levels of MDR1 and MRP1 no matter compared with BGC823 cells or CD44(-) cells (*P* < 0.05) (Fig. 6B). These results revealed that BGC823/5–Fu-CSCs cells had stronger drug resistant ability than CD44(−) cells and they gained resistance to 5-Fu through over-expression of multiple drug-resistant genes in the ABC transporter pathway.

To analyze the relationship between the drug resistance genes and the clinical outcome in GC patients, the prognostic values of MDR1 and MRP1 were evaluated by Kaplan–Meier Plotter. A total of 875 GC patients were available for the analysis of overall survival (OS). The relatively higher expressions of MDR1 (*HR*: 1.4 [1.17–1.68], log-rank *P* = 0.0017) and MRP1 (*HR*: 1.35 [1.14–1.6], log-rank *P* = 5e–04) were associated with an unfavorable prognostic outcome in GC patients (Fig. 6C). It indicated that inhibiting the expression of MDR1 and MRP1 was a potential approach to improve the GC prognosis.

### YQHY efficiently inhibited the drug resistance of BGC823/5–Fu-CSCs

**Fig. 7:**
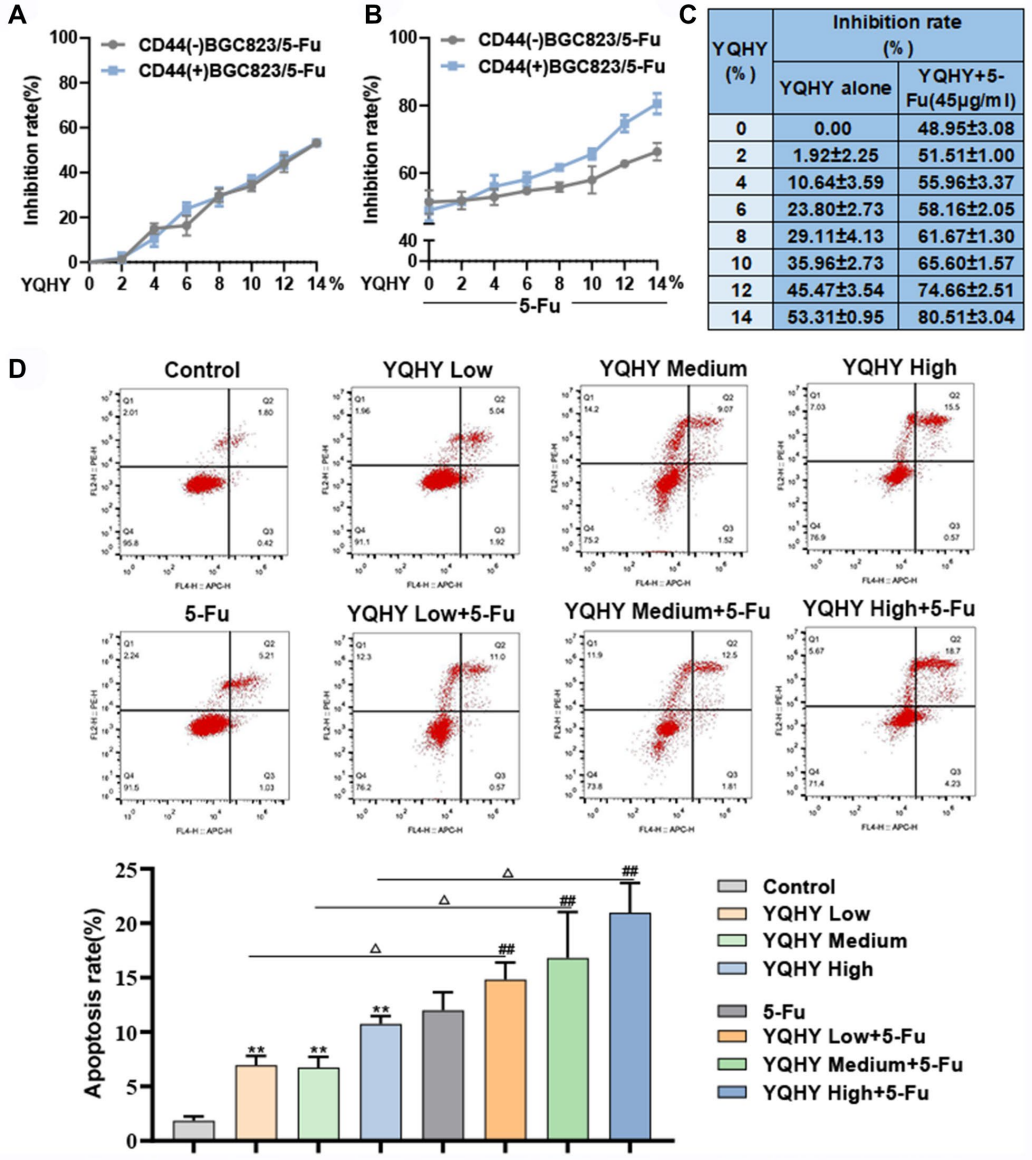
The effect of YQHY on the drug resistance of CD44(-) and CD44(+) (CSCs) cells. **A** The inhibition rate of CD44(−)BGC823/5-Fu and CD44(+)BGC823/5-Fu treated with YQHY. **B** The inhibition rate of CD44(-)BGC823/5-Fu and CD44(+)BGC823/5-Fu treated with YQHY + 5-Fu. **C** The comparison of the inhibition rate of BGC823/5–Fu-CSCs intervened with YQHY or YQHY + 5-Fu. **D** The apoptosis rate of BGC823/5–Fu-CSCs treated with different concentrations of YQHY or combined with 5-Fu. **A**–**D** The duration of administration was 48 h. **B**–**D** The concentration of 5-Fu used in CD44(−)BGC823/5-Fu and CD44(+)BGC823/5-Fu was 20 μg/ml and 45 μg/ml, respectively (the IC50 to 5-Fu of each cell line). * represented the comparison between YQHY and Control group; # represented the comparison between YQHY + 5-Fu and 5-Fu group. ^△^ represented the comparison between YQHY and YQHY + 5-Fu group

To explore the inhibitory effect of YQHY on the drug resistance of CD44(−)BGC823/5-Fu and CD44(+)BGC823/5-Fu, the CCK-8 assay was applied to detect the inhibition rate of the cells after intervention with YQHY combined with 5-Fu (or YQHY alone). As shown in Fig. 7A, B, YQHY alone or YQHY + 5-Fu can both inhibit the growth of CD44(−) and CD44( +) cells. And YQHY combined with 5-Fu had a stronger inhibitory effect. Interestingly, there was no difference in the growth inhibition effect of YQHY alone on the two cells, but when YQHY was administered in combination with 5-Fu, YQHY + 5-Fu had a stronger toxic effect on CD44( +) cells than on CD44(−) cells. This indicated that YQHY had a better drug resistance inhibitory effect on CD44( +) cells than on CD44(-) cells. In addition, under the same intervention of 45ug/ml 5-Fu (the IC50 of 5-Fu to BGC823/5–Fu-CSCs), the inhibition rate of cell proliferation raised gradually with the increase concentration of YQHY (Fig. 7C).

Since apoptosis is an important way in which 5-Fu induces the cell death, we further verified the above results from the perspective of the cell apoptosis. We selected 4%, 8% and 12% as the low, medium and high concentration of YQHY for the further researches. Flow cytometry was used to detect the apoptosis of BGC823/5–Fu-CSCs. With the increase of YQHY concentration, the apoptosis rate of BGC823/5–Fu-CSCs increased gradually. And when intervened with the same concentration of YQHY, the pro-apoptotic ability of YQHY combined with 5-Fu was significantly better than that of YQHY alone (^△^*P* < 0.05) (Fig. 7D). These results demonstrated that YQHY can efficiently reverse the drug resistance of BGC823/5–Fu-CSCs to 5-Fu.

### YQHY suppressed the expression of ABC transporter genes

**Fig. 8:**
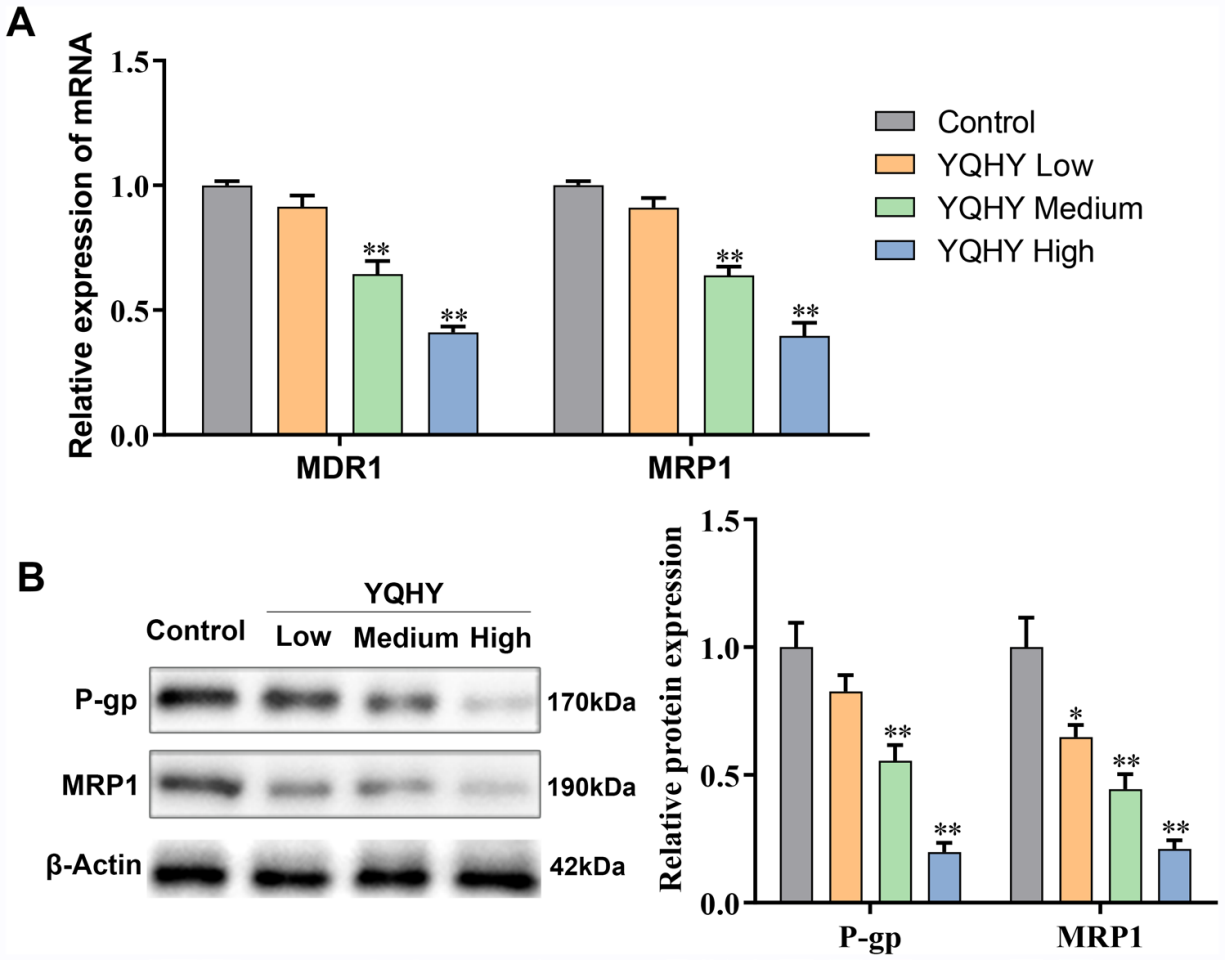
The effect of YQHY on the expression of MDR1 and MRP1. The gene (**A**) and protein (**B**) expression of MDR1 and MRP1 after incubation with YQHY for 48 h

Based on the above experiments, we have found that BGC823/5-Fu-CSCs were characterized with the high expression of MDR1 and MRP1 and YQHY effectively reversed the drug resistance of BGC823/5-Fu-CSCs cells. We envisioned whether YQHY can suppress the ABC transporter pathway to inhibit the drug resistance of BGC823/5-Fu-CSCs. We detected the effects of YQHY on the expression of MDR1 and MRP1 in BGC823/5–Fu-CSCs. Q-PCR demonstrated that the mRNA expression levels of MDR1 and MRP1 were obviously reduced in a YQHY concentration-dependent manner (Fig. 8A). Western blot also showed that the protein expression levels of P-gp (encoded by the MDR1 gene) and MRP1 were decreased (Fig. 8B). These results confirmed that YQHY can restrain the expression of ABC transporter genes which were associated with the drug resistance of BGC823/5–Fu-CSCs.

### YQHY restrained the activation of PI3K/Akt/Nrf signaling pathway in BGC823/5–Fu-CSCs

**Fig. 9:**
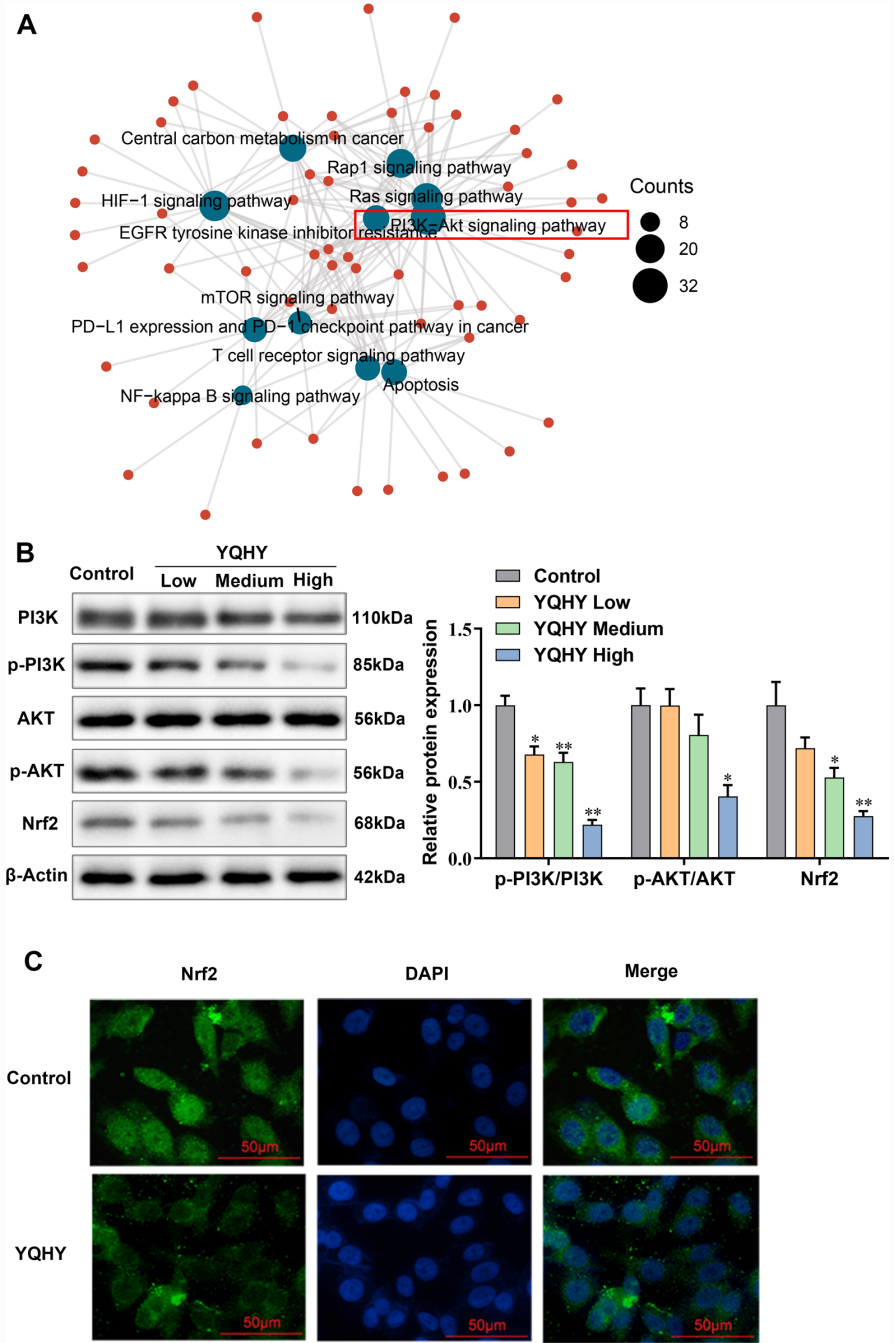
The inhibitory effect of YQHY on the activation of the PI3K/Akt/Nrf2 pathway. **A** KEGG analysis of Yi-qi-hua-yu-jie-du decoction for GC treatment. **B** The expression of PI3K, p-PI3K, AKT, p-AKT and Nrf2 proteins after YQHY intervention for 48 h. **C** Immunofluorescence staining images of Nrf2 after YQHY (the high concentration) intervention for 48 h

We explored the relevant molecular mechanism of YQHY in the inhibition of BGC823/5–Fu-CSC drug resistance. In our previous studies, we used mass spectrometry to analyze the chemical substances of Yi-qi-hua-yu-jie-du decoction and identified the core targets of this formula in GC treatment. KEGG enrichment analysis was performed on the targets of Yi-qi-hua-yu-jie-du decoction in GC treatment. The PI3K/Akt pathway was an important one in the enrichment analysis (Fig. 9A). Current studies have reported that Nrf2 can be a downstream factor of PI3K/AKT and is closely related to the expression of MDR1 and MRP1 [23, 24], so we explored the influence of YQHY on the PI3K/Akt/Nrf2 signaling pathway.

After YQHY intervention on BGC823/5–Fu-CSCs for 48 h, the changes in PI3K, p-PI3K, AKT, p-AKT and Nrf2 were detected by Western blot. It was found that p-PI3K/PI3K, p-AKT/AKT and Nrf2 expression decreased in a YQHY concentration dependent manner, indicating that YQHY can suppress the phosphorylation of the PI3K/AKT pathway and inhibit the expression of Nrf2 (Fig. 9B). The localization of Nrf2 was essential to its biological function, so we further tested the distribution of Nrf2 in cells by immunofluorescence staining. The results demonstrated that YQHY (the high concentration) reduced the fluorescence intensity of Nrf2 in the nucleus and it can restrain the entry of Nrf2 into the nucleus. (Fig. 9C).

### YQHY inhibited the drug resistance of BGC823/5–Fu-CSCs via PI3K/Akt/Nrf2 signaling pathway

**Fig. 10:**
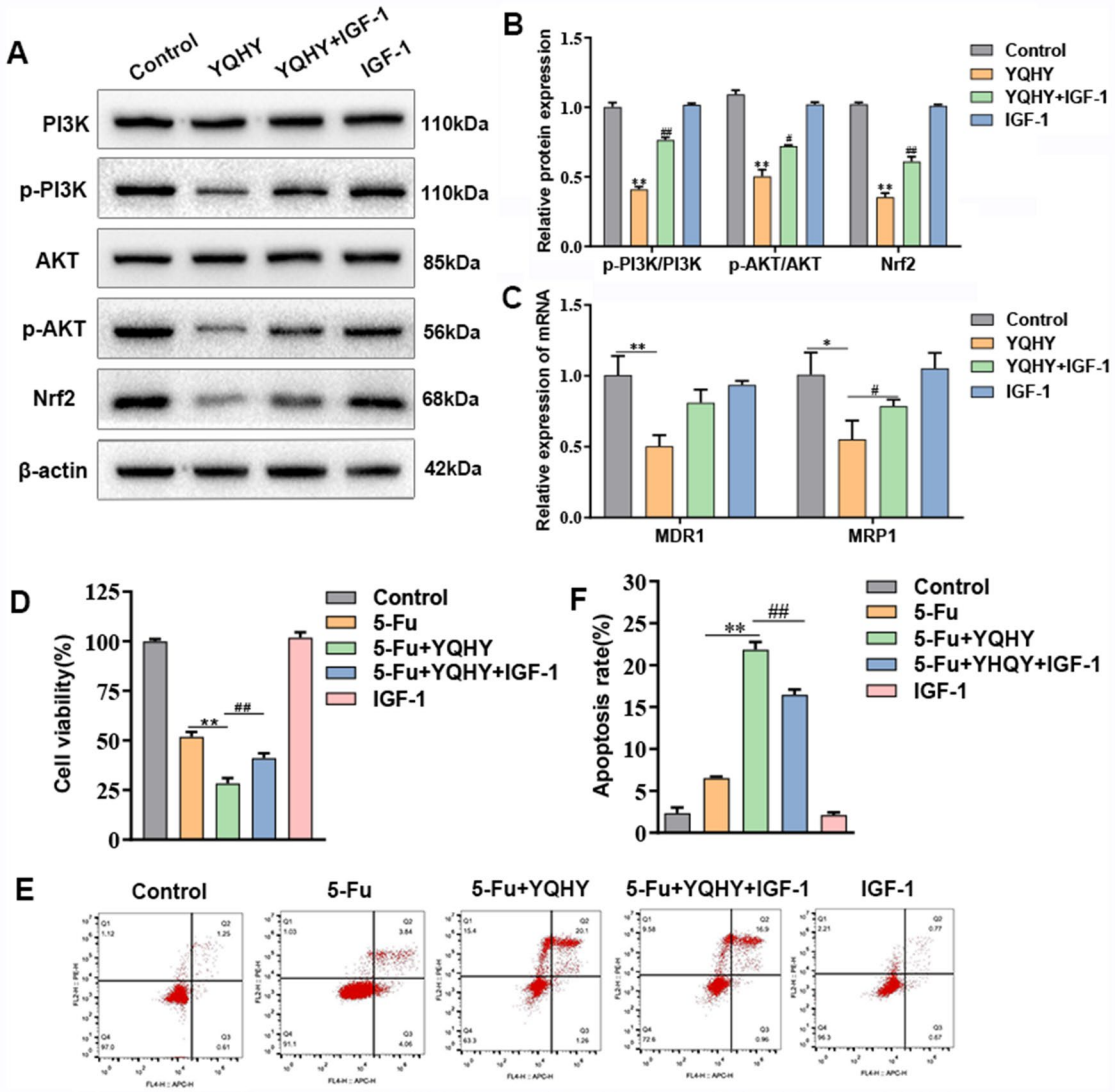
The validation of the mechanism of YQHY on the BGC823/5–Fu-CSCs drug resistance using IGF-1. **A**, **B** The expression of PI3K, p-PI3K, AKT, p-AKT and Nrf2 proteins after YQHY/YQHY + IGF-1 intervention. **C** The mRNA expression of MDR1 and MRP1. **D** The cell viability rates of BGC823/5–Fu-CSCs. **E**, **F** The apoptosis rates of BGC823/5–Fu-CSCs. **A**–**E** The duration of administration was 48 h

To further prove that YQHY suppressed the expression of MDR1 and MRP1 via PI3K/Akt/Nrf2 pathway, thereby reversing the drug resistance of BGC823/5–Fu-CSCs, we intervened BGC823/5–Fu-CSC cells with YQHY (high concentration) and IGF-1(the activator of PI3K/Akt pathway), and observed the changes in PI3K/Akt/Nrf2 pathway, the gene expressions of MDR1 and MRP1, and the drug resistance status of BGC823/5–Fu-CSCs. The results showed that IGF-1 alleviated the inhibitory effect of YQHY on PI3K/AKT/Nrf2 signaling pathway (Fig. 10A, 10B). Meanwhile, it can partially restore the expression levels of MDR1 and MRP1 (Fig. 10C), which confirming that the suppressive effect of YQHY on MDR1 and MRP1 was regulated by the PI3K/AKT/Nrf2 pathway. We further explored the role of PI3K/AKT/Nrf2 pathway in the regulation of YQHY on BGC823/5–Fu-CSCs drug resistance. As shown in Fig. 10D, IGF-1 partially blocked the proliferation-inhibitory effect of YQHY on BGC823/5–Fu-CSCs. Similarly, the promoting apoptosis effect of YQHY was significantly reduced (Fig. 10E, 10F).

**Fig. 11:**
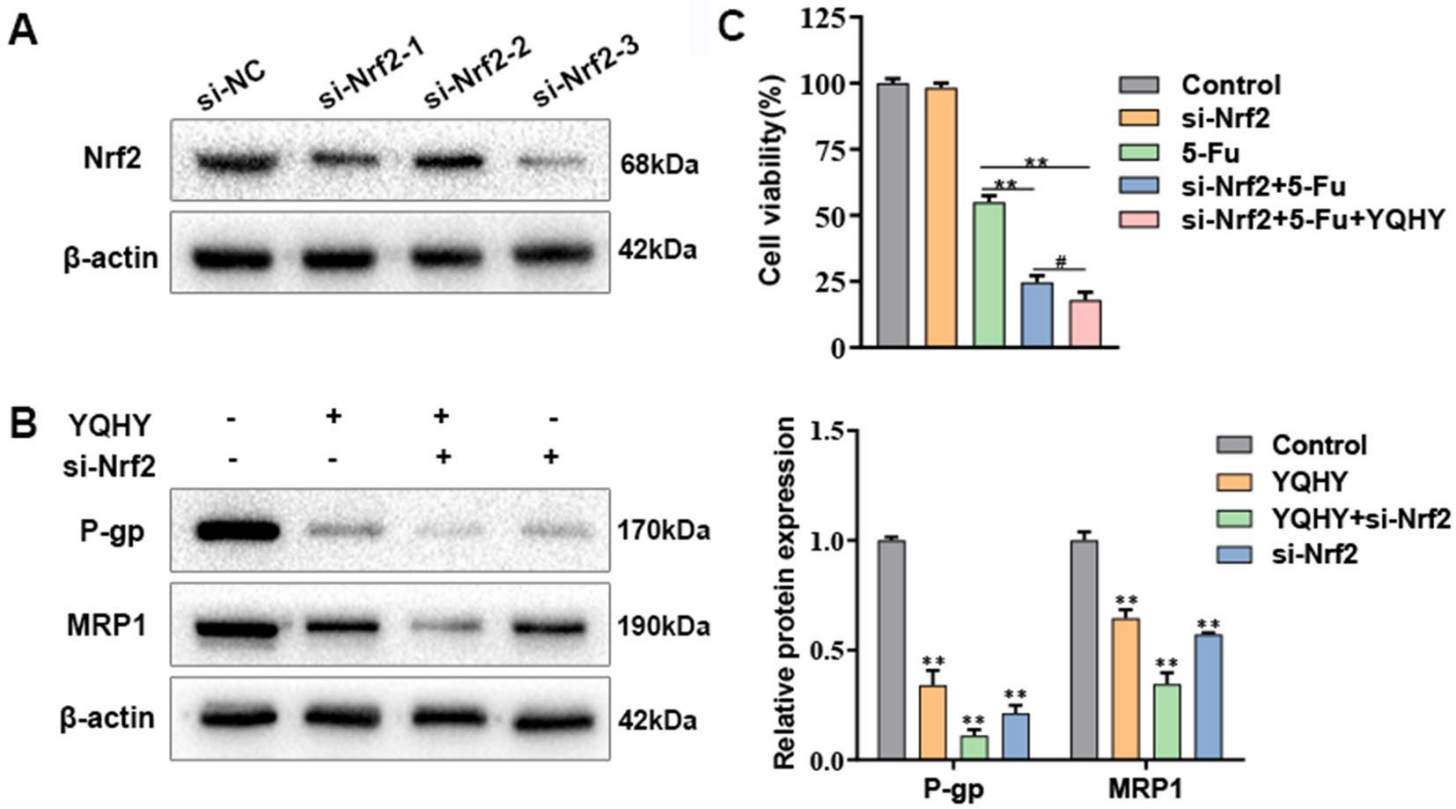
The expression of MDR1 and MRP1 and the sensitivity to 5-Fu in BGC823/5–Fu-CSCs when knockdown of Nrf2. **A** The expression of Nrf2 after intervened with Nrf2 siRNA. The si-Nrf2-3 gained the most Nrf2 knockdown efficiency and it was used in the subsequent experiments. **B** The expression of P-gp (encoded by MDR1) and MRP1. **C** The cell viability of BGC823/5–Fu-CSCs. **B**, **C** The duration of administration was 48 h

In addition, we applied siRNA assay to gain a Nrf2-knockdown BGC823/5-Fu-CSCs cell line (Fig. 11A, si-Nrf2-3). It was found that si-Nrf2 can reduce the expression of P-gp and MRP1, while the combination of YQHY and si-Nrf2 had a stronger inhibitory effect on these drug resistant proteins (Fig. 11B). Similarly, si-Nrf2 + 5-Fu can produce high cytotoxicity to BGC823/5-Fu-CSCs, and YQHY + siNrf2 + 5-Fu presented the most significant inhibition on the cell viability (Fig. 11C). This indicated that Nrf2 was a vital target in the drug resistance of BGC823/5–Fu-CSCs. YQHY and si-Nrf2 synergistically increased the suppressive effect of the drug resistance in BGC823/5–Fu-CSCs.

These results verified that YQHY inhibited the MDR1 and MRP1 expression via PI3K/Akt/Nrf2, thus reversing the drug resistance of BGC823/5–Fu–CSCs.

## Discussion

Drug resistance in gastric cancer is a complex process involving multiple factors, including cell membrane transporter abnormalities, increased DNA damage repair, decreased apoptosis, epithelial-mesenchymal transition, changes in the activity of enzymes in cells and disorder of microRNA regulation [25]. As the root cause of tumor formation and recurrence, CSCs are closely related to tumor invasion, metastasis, chemotherapy resistance and recurrence. Existing research evidences showed that drug resistance is inseparable from the existence of stem cells in gastric cancer [26], and CSCs featured with over-expression of ABC transporters are one of important reasons for chemotherapy resistance [27]. Therefore, it is of great significance to explore the mechanism of stem cell drug resistance and seek out drug resistance reversal agents to improve the effectiveness of clinical treatment of gastric cancer.

Yi-qi-hua-yu-jie-du decoction was derived from the classic traditional Chinese medicine prescription "Gui-Shao-Liu-Jun-Zi Decoction". A multi-center and large-sample clinical study (NO. 200807022) funded by the National Administration of Traditional Chinese Medicine showed that it can effectively reduce the recurrence rate of stage II and III postoperative GC patients combined with chemotherapy. The recurrence risk was reduced by 32.8% compared with chemotherapy alone [15]. In the previous studies, our research team found that Jianpi Yangwei decoction (the forerunner of Yi-qi-hua-yu-jie-du decoction) inhibited the proliferation of BGC823/5-Fu cells and reversed gastric cancer drug resistance via the PI3K/AKT signaling pathway [17]. It revealed that targeting drug resistance may be one of the mechanisms for this prescription playing an effective role in clinical practice. In this study, the drug-resistant stem cell model CD44(+)BGC823/5-Fu was constructed, and the stemness characteristics were identified from multi-aspects. Yi-qi-hua-yu-jie-du medicated serum can suppress the expression of MDR1/P-gp and MRP1 in BGC823/5–Fu-CSCs, thereby effectively reducing GC drug resistance. Finally, based on the KEGG analysis of Yi-qi-hua-yu-jie-du decoction in gastric cancer treatment and combined with the using of IGF-1 and si-Nrf2, we found that YQHY prevented the activation of the PI3K/Akt/Nrf2 pathway thus to restrain the drug resistance of BGC823/5–Fu-CSCs. This study further revealed a new mechanism of Yi-qi-hua-yu-jie-du decoction on the suppression of GC stem cells drug resistance based on ABC transporter pathway. Since BGC823/5-Fu-CSCs were stem cells isolated from BGC823/5-Fu with CD44(+) as a biomarker, we found that BGC823/5-Fu-CSC exhibited stronger proliferative and tumorigenic abilities while preserving the drug resistance characteristics. Therefore, intervention of CSCs was a more efficient way to reverse the drug resistance and inhibit the proliferation of gastric cancer. In our study, although BGC823/5-Fu-CSCs presented the higher expression levels of MDR1 and MRP1, it was more sensitive to the intervention of YQHY combined with 5-Fu, indicating that CSCs was the dominant cell group of YQHY in the treatment of reversing gastric cancer drug resistance. And the reason for this phenomenon may be related to the inhibitory effect of YQHY on MDR1 and MRP1. It was not only a deepening of previous studies but also a further exploration of the experimental basis for Yi-qi-hua-yu-jie-du decoction in gastric cancer treatment. More importantly, based on the correlation between ABC transporters and poor GC clinical prognosis, inhibiting the expression of MDR1 and MRP1 in CSCs may be a potential direction for improving GC prognosis.

As the root of tumor occurrence and development, the concept of tumor stem cell has brought dawn to the treatment evolution of tumors. CSCs are characterized by the features of DNA repair function activation and apoptosis resistance, etc., leading to insensitivity to most current anti-mitotic chemotherapy drugs [28]. The existence of drug-resistant stem cells is one of the important reasons for tumor chemotherapy failure. The high expression of the ABC transporter genes is a crucial mechanism to prevent CSCs from chemotherapy toxicity. This makes CSCs incipiently resistant to various chemotherapies [29]. These transmembrane proteins constitute a drug delivery pump system that can expel drugs from cells in an energy-dependent manner, limiting the drug concentrations inside cells and weakening the efficacy of chemotherapy drugs. ABC transporters are classic targets of drug resistance inhibitors, and three generations of inhibitors have been developed, including verapamil, dexverapamil, tariquidar and elacridar [30]. First-generation inhibitors represented by verapamil need a high concentration to inhibit P-gp expression in clinical use. Although dexverapamil and other second-generation inhibitors have stronger inhibitory effects on P-gp, their high affinity with CYP450 limits their clinical application. The current development of third-generation inhibitors aim at identifying substances with high specificity and low toxicity [31]. However, these inhibitors can hardly pass clinical trials due to their strong side effects or actually poor efficacy [32], so finding suitable ABC transporter inhibitors has become a research hotspot in reversing drug resistance of tumor.

Recently, many studies have found a variety of traditional Chinese medicine substances can reduce tumor drug resistance via ABC transporters [33]. And this shifted our attention to finding effective drug resistance inhibitors from natural medicines. 4-Hydroxyemodin [34] can inhibit P-gp activity and inhibit the proliferation and migration of paclitaxel-resistant cells through the AKT/ERK pathway. 7-O-geranylquercetin [35] can suppress the expression level of P-gp and its encoding gene MDR1 in drug-resistant cells, thus inhibiting the proliferation of adriamycin resistant breast cancer cells. Other substances, such as curcumin, ligustrazine, dibenzocyclooctadiene lignans, and coumarins, also inhibit tumor drug resistance by regulating ABC transporters [36]. However, at the same time, there are few studies on Chinese herbal compounds, which are most widely used in clinical. Therefore, we conducted the above study to explore the effect of Yi-qi-hua-yu-jie-du decoction on ABC transporter mediated drug resistance of gastric cancer stem cells to strengthen the experimental theoretical basis of TCM in anti-gastric cancer drug resistance. And these findings will be the direction of our future research to further reveal the effective role of Yi-qi-hua-yu-jie-du decoction in GC treatment.

## Conclusion

This study successfully constructed a CD44( +) drug resistant GC stem cell model and found that ABC transporter genes MDR1 and MRP1 were up-regulated in BGC823/5–Fu-CSCs. The TCM compound Yi-qi-hua-yu-jie-du decoction can reverse the drug resistance of BGC823/5–Fu-CSCs by suppressing MDR1 and MRP1 via the PI3K/Akt/Nrf2 pathway.

## Supplementary Information

**Additional file 1: Supplementary Table 1**. The name of 15 Chinese herbal medicine in Yi-qi-hua-yu-jie-du decoction.** Supplementary Table 2**. The Primer sequences of the genes detected by Q-PCR.** Supplementary Figure 1**. The construction of co-expression modules by WGCNA.(A)The incomplete data sets were excluded (over the red line). (B)The heatmap of theoverview in the mRNAsi values and the EREG-mRNAsi values. (C) The appropriatepower value 4 was selected for the consideration of the scale-free correlationcoefficient and mean connectivity. (D)The GeneTree was constructed based on thepower value. (E)The module similarity was calculated to join the modules.

## Data Availability

The raw data of this article will be made available by the authors to any qualified researcher.

## Abbreviations

TCM: Traditional Chinese Medicine
GC: Gastric cancer
YQHY: Yi-qi-hua-yu-jie-du medicated serum
MACS: Magnetic-activated cell sorting
CSCs: Cancer stem cells
ABC transporters: ATP2 binding cassette transporters
NANOG: Nanog homeobox
OCT4: Octamer-binding transcription factor 4
SOX2: SRY-box transcription factor 2
MDR1: Multidrug resistance gene 1
MRP1: Multidrug resistance associate protein 1
SFM: Serum-free medium
HPLC: High Performance Liquid Chromatography
DEGs: Differentially expressed genes
WGCNA: Weighted gene coexpression network analysis

## References

[1] Bray F, Ferlay J, Soerjomataram I, Siegel RL, Torre LA, Jemal A (2018). Global cancer statistics 2018: GLOBOCAN estimates of incidence and mortality worldwide for 36 cancers in 185 countries. CA Cancer J Clin.

[2] Lytle NK, Barber AG, Reya T (2018). Stem cell fate in cancer growth, progression and therapy resistance. Nat Rev Cancer.

[3] Moore MA, Williams N, Metcalf D (1973). In vitro colony formation by normal and leukemic human hematopoietic cells: characterization of the colony-forming cells. J Natl Cancer Inst.

[4] Lapidot T, Sirard C, Vormoor J (1994). A cell initiating human acute myeloid leukaemia after transplantation into SCID mice. Nature.

[5] Chae YC, Kim JH (2018). Cancer stem cell metabolism: target for cancer therapy. BMB Rep.

[6] Zhu P, Fan Z (2018). Cancer stem cells and tumorigenesis. Biophys Rep.

[7] Schoning JP, Monteiro M, Gu W (2017). Drug resistance and cancer stem cells: the shared but distinct roles of hypoxia-inducible factors HIF1alpha and HIF2alpha. Clin Exp Pharmacol Physiol.

[8] Fu Y, Du P, Zhao J, Hu C, Qin Y, Huang G (2018). Gastric cancer stem cells: mechanisms and therapeutic approaches. Yonsei Med J.

[9] Alisi A, Cho WC, Locatelli F, Fruci D (2013). Multidrug resistance and cancer stem cells in neuroblastoma and hepatoblastoma. Int J Mol Sci.

[10] Saini N, Yang X (2018). Metformin as an anti-cancer agent: actions and mechanisms targeting cancer stem cells. Acta Biochim Biophys Sin (Shanghai).

[11] Zhu X, Tao X, Lu W, Ding Y, Tang Y (2019). Blockade of integrin beta3 signals to reverse the stem-like phenotype and drug resistance in melanoma. Cancer Chemother Pharmacol.

[12] Liu RM, Xu P, Chen Q, Feng SL, Xie Y (2020). A multiple-targets alkaloid nuciferine overcomes paclitaxel-induced drug resistance in vitro and in vivo. Phytomedicine.

[13] Zhu GX, Gao D, Shao ZZ, Chen L, Ding WJ, Yu QF (2021). Wnt/betacatenin signaling: causes and treatment targets of drug resistance in colorectal cancer (Review). Mol Med Rep.

[14] Suzuki S, Okada M, Sanomachi T (2020). Therapeutic targeting of pancreatic cancer stem cells by dexamethasone modulation of the MKP-1-JNK axis. J Biol Chem.

[15] Shu P, Tang H, Zhou B (2019). Effect of Yiqi Huayu Jiedu decoction on stages II and III gastric cancer: a multicenter, prospective, cohort study. Medicine.

[16] Huang W, Tang H, Wen F, Lu X, Li Q, Shu P (2020). Jianpi-yangwei decoction inhibits DNA damage repair in the drug resistance of gastric cancer by reducing FEN1 expression. BMC Complement Med Ther.

[17] Tang H, Huang W, Yang Q, Lin Y, Chen Y, Shu P (2020). Jianpi Yangwei decoction promotes apoptosis and suppresses proliferation of 5-fluorouracil resistant gastric cancer cells in vitro and in vivo. BMC Complement Med Ther.

[18] Phi LTH, Sari IN, Yang YG (2018). Cancer stem cells (CSCs) in drug resistance and their therapeutic implications in cancer treatment. Stem Cells Int.

[19] Wang J, Li X, Wu H (2019). EMP1 regulates cell proliferation, migration, and stemness in gliomas through PI3K-AKT signaling and CD44. J Cell Biochem.

[20] Wang L, Zuo X, Xie K, Wei D (2018). The role of CD44 and cancer stem cells. Methods Mol Biol.

[21] Pothuraju R, Rachagani S, Krishn SR (2020). Molecular implications of MUC5AC-CD44 axis in colorectal cancer progression and chemoresistance. Mol Cancer.

[22] Sun L, Fang Y, Wang X (2019). miR-302a inhibits metastasis and cetuximab resistance in colorectal cancer by targeting NFIB and CD44. Theranostics.

[23] Qiu L, Ma Z, Li X (2020). DJ-1 is involved in the multidrug resistance of SGC7901 gastric cancer cells through PTEN/PI3K/Akt/Nrf2 pathway. Acta Biochim Biophys Sin (Shanghai).

[24] Li J, Wang T, Liu P (2021). Hesperetin ameliorates hepatic oxidative stress and inflammation via the PI3K/AKT-Nrf2-ARE pathway in oleic acid-induced HepG2 cells and a rat model of high-fat diet-induced NAFLD. Food Funct.

[25] Huang WJ, Ruan S, Wen F (2020). Multidrug resistance of gastric cancer: the mechanisms and Chinese medicine reversal agents. Cancer Manag Res.

[26] Li K, Dan Z, Nie YQ (2014). Gastric cancer stem cells in gastric carcinogenesis, progression, prevention and treatment. World J Gastroenterol.

[27] Lo YL, Liu Y (2014). Reversing multidrug resistance in Caco-2 by silencing MDR1, MRP1, MRP2, and BCL-2/BCL-xL using liposomal antisense oligonucleotides. PLoS ONE.

[28] Wang T, Shigdar S, Gantier MP (2015). Cancer stem cell targeted therapy: progress amid controversies. Oncotarget.

[29] McIntosh K, Balch C, Tiwari AK (2016). Tackling multidrug resistance mediated by efflux transporters in tumor-initiating cells. Expert Opin Drug Metab Toxicol.

[30] Beretta GL, Cassinelli G, Pennati M, Zuco V, Gatti L (2017). Overcoming ABC transporter-mediated multidrug resistance: the dual role of tyrosine kinase inhibitors as multitargeting agents. Eur J Med Chem.

[31] Dewanjee S, Dua TK, Bhattacharjee N (2017). Natural products as alternative choices for P-glycoprotein (P-gp) inhibition. Molecules.

[32] Waghray D, Zhang Q (2018). Inhibit or evade multidrug resistance P-glycoprotein in cancer treatment. J Med Chem.

[33] Lou JS, Yao P, Tsim KWK (2018). Cancer treatment by using traditional Chinese medicine: probing active compounds in anti-multidrug resistance during drug therapy. Curr Med Chem.

[34] Lin H, Hu B, He X (2020). Overcoming Taxol-resistance in A549 cells: a comprehensive strategy of targeting P-gp transporter, AKT/ERK pathways, and cytochrome P450 enzyme CYP1B1 by 4-hydroxyemodin. Biochem Pharmacol.

[35] Zhang E, Liu J, Shi L (2019). 7-O-geranylquercetin contributes to reverse P-gp-mediated adriamycin resistance in breast cancer. Life Sci.

[36] Ma X, Hu M, Wang H, Li J (2018). Discovery of traditional Chinese medicine monomers and their synthetic intermediates, analogs or derivatives for battling P-gp-mediated multi-drug resistance. Eur J Med Chem.

