# Supplementary material for: The inhibitory effect and mechanism of Yi-qi-hua-yu-jie-du decoction on the drug resistance of gastric cancer stem cells based on ABC transporters
Source: Chin Med. 2022 Aug 9;17:93. doi: 10.1186/s13020-022-00647-y (PMC9361523; doi:10.1186/s13020-022-00647-y)
Supplement: Supplementary file 1 — Additional file 1: Supplementary Table 1. The name of 15 Chinese herbal medicine in Yi-qi-hua-yu-jie-du decoction. Supplementary Table 2. The Primer sequences of the genes detected by Q-PCR. Supplementary Figure 1. The construction of co-expression modules by WGCNA.(A)The incomplete data sets were excluded (over the red line). (B)The heatmap of theoverview in the mRNAsi values and the EREG-mRNAsi values. (C) The appropriatepower value 4 was selected for the consideration of the scale-free correlationcoefficient and mean connectivity. (D)The GeneTree was constructed based on thepower value. (E)The module similarity was calculated to join the modules. [file 13020_2022_647_MOESM1_ESM.pdf]

**Supplementary Table 1**

The name of 15 Chinese herbal medicine in Yi-qi-hua-yu-jie-du decoction.

| Scientific name* | Name in Chinese |
|---|---|
| *Astragalus mongholicus Bunge* | Huang-qi |
| *Codonopsis pilosula (Franch.) Nannf.* | Dang-shen |
| *Citrus × acida Pers.* | Chen-pi |
| *Pinellia tuberifera Ten.* | Ban-xia |
| *Poria cocos* | Fu-ling |
| *Aucklandia costus Falc.* | Mu-xiang |
| *Wurfbainia uliginosa (J.Koenig) Giseke* | Sha-ren |
| *Atractylodes macrocephala Koidz* | Bai-zhu |
| *Paeonia lactiflora Pall.* | Bai-shao |
| *Angelica sinensis (Oliv.) Diels* | Dang-gui |
| *Sparganium stoloniferum (Buch.-Ham. ex Graebn.) Buch.-Ham. ex Juz.* | San-leng |
| *Curcuma aromatica Salisb* | E-zhu |
| *Salvia chinensis Benth.* | Shi-jian-chuan |
| *Scleromitrion angustifolium (Cham. & Schltdl.) Benth.* | Bai-hua-she-she-cao |
| *Glycyrrhiza glabra L.* | Gan-cao |

* The name validated in www.theplantlist.org.

**Supplementary Table 2**

The Primer sequences of the genes detected by Q-PCR.

β-actin-f (5-CAGTCGGTTGGAGCGAGCAT-3);

β-actin-r (5-GGACTTCCTGTAACAACGCATCT-3);

NANOG-f (5-AAGGTCCCGGTCAAGAAACAG-3);

NANOG-r (5-CTTCTGCGTCACACCATTGC-3);

OCT4-f (5-CTGGGTTGATCCTCGGACCT-3);

OCT4-r (5-CCATCGGAGTTGCTCTCCA-3);

SOX2-f (5-GCCGAGTGGAAACTTTTGTCG-3);

SOX2-r (5-GGCAGCGTGTACTTATCCTTCT-3);

MDR1-f (5-CAACGCATTGCCATAGCTC-3);

MDR1-r (5-AGGGCTTCTTGGACAACCT-3);

MRP1-f (5-CAACGCATTGCCATAGCTC-3);

MRP1-r (5-GCCTCATCCAACACAAGGAT-3);

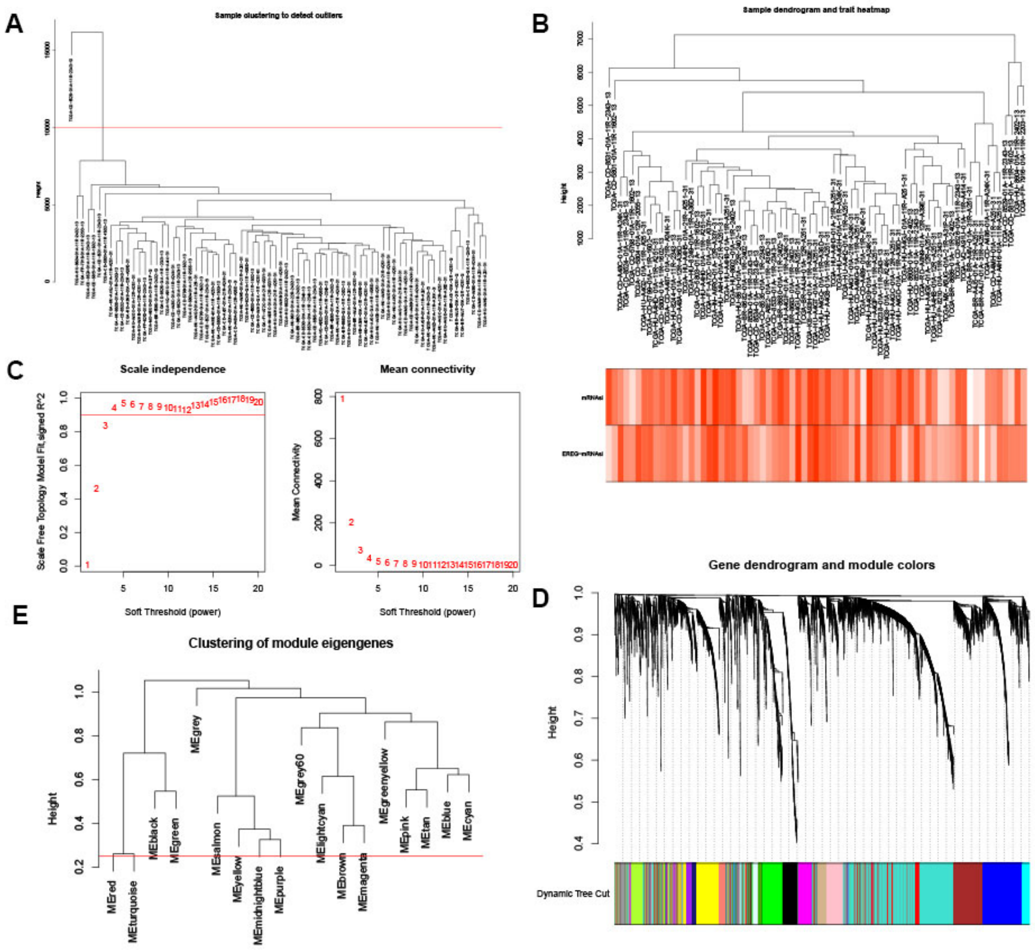


**Supplementary Figure 1.** The construction of co-expression modules by WGCNA. (A)The incomplete data sets were excluded (over the red line). (B)The heatmap of the overview in the mRNAsi values and the EREG-mRNAsi values. (C) The appropriate power value 4 was selected for the consideration of the scale-free correlation coefficient and mean connectivity. (D)The GeneTree was constructed based on the power value. (E)The module similarity was calculated to join the modules.
